# Antitumor activity of a *Trans*-thiosemicarbazone schiff base palladium (II) complex on human gastric adenocarcinoma cells

**DOI:** 10.18632/oncotarget.14620

**Published:** 2017-01-13

**Authors:** Bingchang Zhang, Haiqing Luo, Qinjuan Xu, Lirong Lin, Bing Zhang

**Affiliations:** ^1^ College of Chemistry and Chemical Engineering, University of Xiamen, Xiamen, 361005, P. R. China; ^2^ School of Medicine, University of Xiamen, Xiamen, 361102, P. R. China

**Keywords:** trans-thiosemicarbazone Schiff base palladium (II) complex, human gastric adenocarcinoma cells, a nude mouse tumor xenograft model, antitumor activity

## Abstract

The development of transition-metal-based antitumor drug candidates increases the metallopharmaceuticals study dramatically. Two *trans*-thiosemicarbazone-based, Schiff base palladium (Pd) (II) complexes, DMABTSPd (TSPd) and DMABPTSPd (PTSPd), were prepared and characterized as described in our previous study. Here, we investigated whether the two complexes have antitumor effect on human gastric adenocarcinoma cell lines, BGC-823 and SGC-7901, compared with normal human gastric mucosal epithelial cell line, Ges-1. The results show that the Pd complex with the bare amino group (DMABTSPd(TSPd)) can inhibit cell viabilities and induce apoptosis in human gastric carcinoma cells, rather than the Pd complex without the bare amino group (DMABPTSPd (PTSPd)). This occurs via a mitochondrial-related pathway by down-regulating the level of Bcl-2 expression and up-regulating the level of Bid expression. Meanwhile, DMABTSPd (TSPd) suppressed tumor growth via a mitochondrial-related pathway in a nude mouse tumor xenograft model derived from BGC-823 cells. These findings demonstrate that DMABTSPd (TSPd) is worthy of further structural optimization and representing a promising Pd complex for the development of a new antitumor therapeutic agent.

## INTRODUCTION

The discovery of the antitumor activity of transition-metal-based drug candidates (such as Pt, Pd, and Mo complexes) causes research into metallopharmaceuticals to increase dramatically [1–4]. These metal complexes have good activity towards one or more tumor cell lines, and some have entered clinical trials. However, the major problems with transition-metal-based antitumor drugs are their side effects and cell resistance, varying from nephrotoxicity to drug resistance of the tumor cells, along with limited applicability towards certain cancer cell lines [5, 6]. These have prompted researchers to look for alternative metallopharmaceuticals with improved properties to widen the spectrum of treatable cancers, reduce toxic side effects, and overcome metal resistance.

A series of novel palladium (Pd) complexes have been synthesized that exhibit good activity against tumor cell lines [7–9]. As an example, organometallic Pd complexes with a water-soluble iminophosphorane ligand could be potential anticancer agents [8]. [1-benzyl-3-tert-butylimidazol-2-ylidene] 2PdCl2 shows strong antiproliferative activity against three types of human tumor cells, namely, cervical cancer (HeLa), breast cancer (MCF-7), and colon adenocarcinoma (HCT116), in culture [7]. Especially, the Pd (II) complexes bearing thiosemicarbazone-based Schiff bases have greater antiproliferative activity than thiosemicarbazones, even if thiosemicarbazones have potential therapeutic activity and are widely used in medicine, including LM3 (mammary adenocarcinoma) and LP07 (lung adenocarcinoma) cell lines [10]. In the thiosemicarbazone-based Schiff base Pd (II) complexes, the ligands are usually bonded to the Pd (II) center with an O, N, S-tridentate coordination mode. This tridentate coordination mode stabilizes the Pd (II) complexes and prevents possible *cis-trans* isomerization to give a more positive effect [11]. The tridentate coordination mode of the Pd (II) complex is neither *cis*-geometry nor *trans*-geometry. The importance of the *trans*-geometry around the Pd center has been attributed to the comparatively higher cytotoxicity values compared with those for *cis*-isomers. In addition, the bare amino group may play an important role in the differences in cytotoxicity [12], this prompted us to design *trans*-geometry thiosemicarbazone-based Schiff base Pd (II) complexes, with and without bare amino group, to study their cytotoxic effect against a selection of cell lines.

Gastric cancer is the third leading cause of cancer-related deaths with a global burden of 723000 deaths in 2012 [13]. Many patients develop a relapse after curative surgery, in part due to the vacancy of current therapy agents [14]. It is urgent to find more effective therapeutic drugs for gastric cancer therapy.

**Figure 1:**
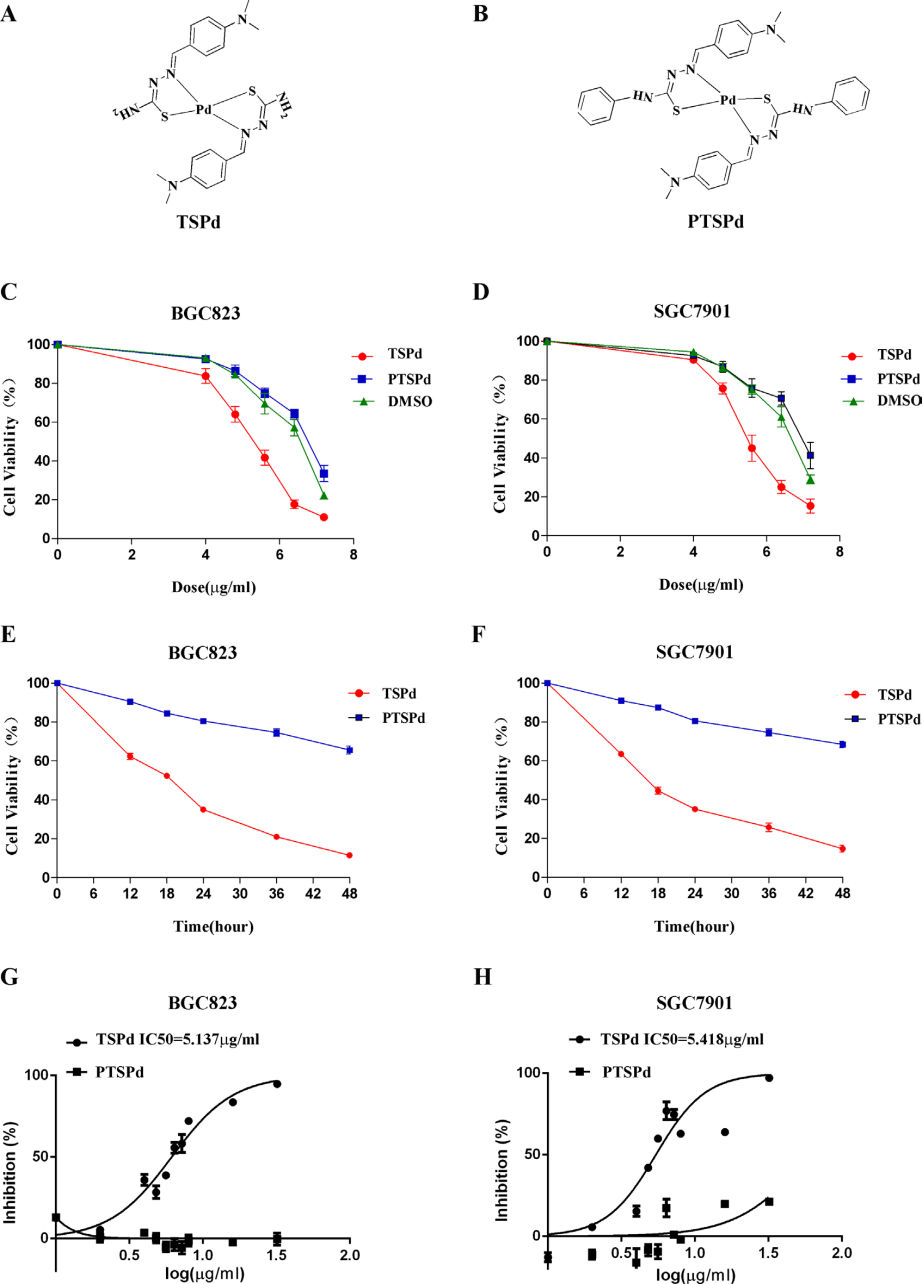
Effect of the target complexes on cell viabilities of human gastric carcinoma cells **(A, B)** Molecular structures of the two Pd (II) complexes, DMABTSPd(TSPd) and DMABPTSPd (PTSPd). **(C, D)** BGC823 and SGC7901 cells were treated with different doses of target complexes (0, 4.0, 4.8, 5.6, 6.4, and 7.2 μg/mL) and DMSO (the same volume as the highest drug dose). **(E, F)** BGC823 and SGC7901 cells were treated with 6.4 μg/mL target complexes for different time periods (0, 12, 24, 36, and 48 h). The cell viabilities were measured by an MTT assay as described in the Materials and Methods section. The data are representative of three independent experiments, each yielding similar results. G&H: BGC823 and SGC7901 cells were treated with the target complexes or DMSO at the indicated doses, followed by detection of the anticancer activity (IC50 values) as described in the Materials and Methods section. **(G)** The IC50 for DMABTSPd(TSPd) was determined to be 5.137 μg/mL (means ± S.D., n = 6) in BGC-823 cells. **(H)** The IC50 for DMABTSPd (TSPd) was determined to be 5.418 μg/mL (means ± S.D., n = 6) in SGC-7901 cells.

In this study, we investigated the antitumor activity and mechanism of two *trans*-thiosemicarbazone, Schiff base palladium (Pd) complexes DMABTSPd(TSPd), with bare amino group, and DMABPTSPd (PTSPd), without bare amino group (Figure 1A and 1B ) in human gastric adenocarcinoma cell lines, BGC-823 and SGC-7901, *in vitro and in vivo*, compared to normal human gastric mucosal epithelial cell line, Ges-1. In our target complexes, the thiosemicarbazone-based Schiff base Pd (II) complexes, the ligands are bonded to the Pd (II) center with an N, S-bidentate coordination mode. This bidentate coordination mode stabilizes the Pd (II) complexes and prevents possible cis-trans isomerization. The importance of the trans-geometry around the Pd center has been attributed to the comparatively higher cytotoxicity values compared with those for cis-isomers. In addition, the with and without bare amino group might play a different role in cytotoxicity. The data confirm that DMABTSPd(TSPd) is worthy of further structural optimization and representing a promising Pd complex for the development of a new antitumor therapeutic agent.

## RESULTS

### The effect of the two complexes on cell viabilities of human gastric adenocarcinoma cells

BGC823 and SGC7901 cells were treated with different doses of DMABTSPd(TSPd) and DMABPTSPd (PTSPd) for 24 h, respectively, and cell viability of human gastric cancer cells was determined using an MTT assay. Figure 1C and 1D showed that cell viability of the two lines significantly decreased in a DMABTSPd (TSPd) dose-dependent manner, compared with DMSO group. Whereas, the treatment of DMABPTSPd (PTSPd) did not have significant effect on cell viability of the two cell lines, compared with DMSO group (Figure 1C and 1D). Furthermore, after BGC823 and SGC7901 cells were treated with 6.4 μg/mL DMABTSPd (TSPd) or DMABPTSPd (PTSPd) for different time points, cell viability of the two cell lines significantly decreased in the DMABTSPd(TSPd)-treated group in a time-dependent manner, compared with the DMABPTSPd (PTSPd)-treated group (Figure 1E and 1F). Additionally, BGC823 and SGC7901 cells are especially sensitive to DMABTSPd(TSPd) with IC_50_ values of 5.1 μg/mL and 5.4 μg/mL, respectively, compared with the DMABPTSPd(PTSPd)-treated group (Figure 1G and 1H). Therefore, the data indicated that DMABTSPd(TSPd) inhibited cell viability of BGC823 and SGC7901 cells in a dose- and time-dependent manner, while DMABPTSPd (PTSPd) did not. Additionally, we observed that the two complexes had not any effect on the cell viability of normal human gastric mucosal epithelial cell line, Ges-1 (Supplementary Figure 1).

### The effect of the two complexes on cell apoptosis of human gastric carcinoma cells

**Figure 2:**
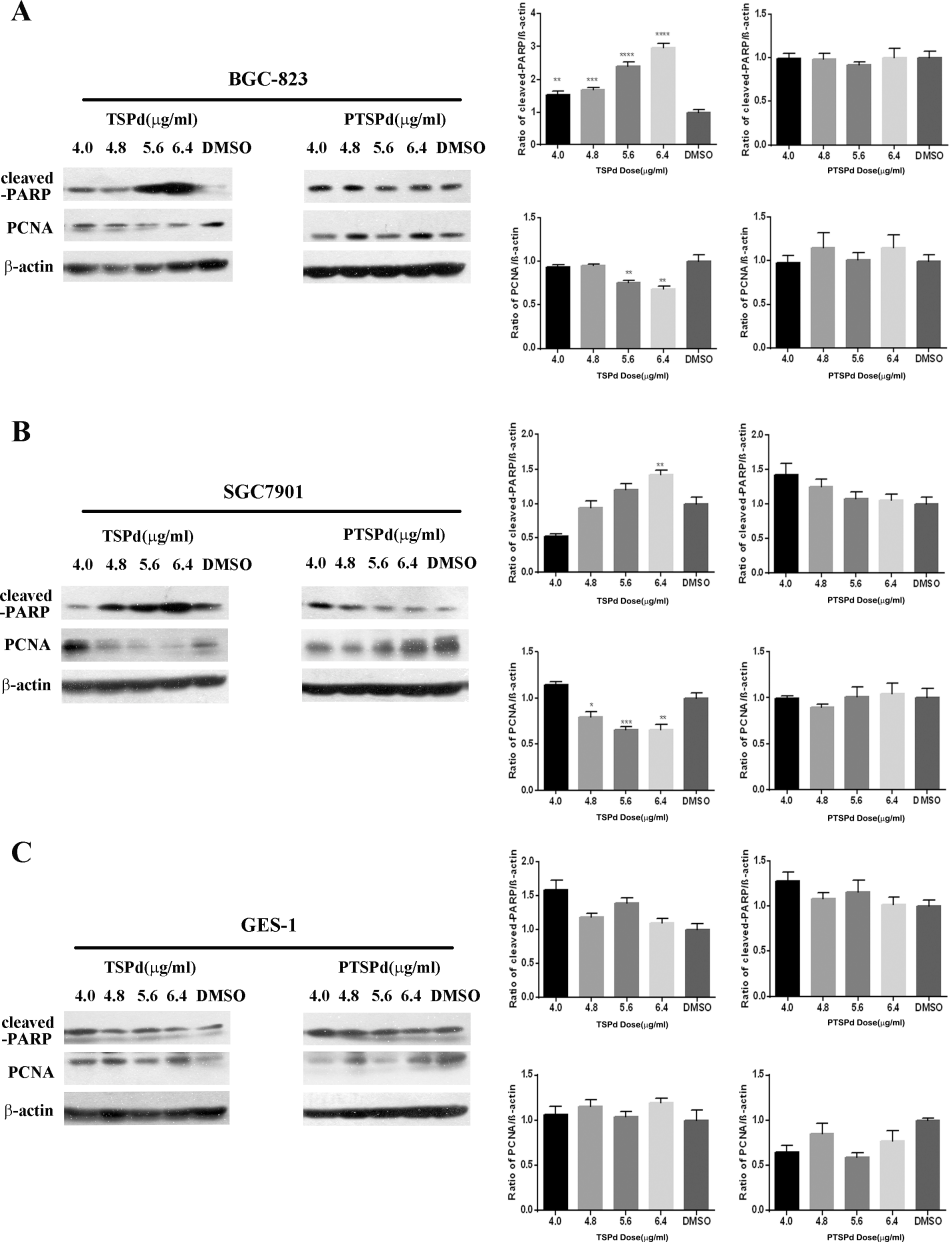
Effect of the target complexes on cell apoptosis of human gastric carcinoma cells and Ges-1 cells Cells were treated with different doses of DMABTSPd(TSPd) and DMABPTSPd (PTSPd) (4.0, 4.8, 5.6, and 6.4 μg/mL) for 24 h and the DMSO group was treated with the same volume of DMSO as the highest dose of the drug treatment group. The levels of cleaved-PARP and PCNA expression were detected by western blotting analysis as described in the Materials and Methods section. **(A)** BGC823 cells. **(B)** SGC7901 cells. **(C)** Ges-1 cells. Data are reported as means ± S.D. of three separate experiments (**P < 0.01, ***P < 0.001, ****P < 0.0001, vs the control group).

After BGC823 and SGC7901 cells were treated with different dose of DMABTSPd(TSPd) or DMABPTSPd (PTSPd) for 24 h, western blotting analysis showed that DMABTSPd(TSPd) promoted the cleavage of poly ADP-ribose polymerase (cleaved-PARP) in BGC823 and SGC7901 cells, compared with DMSO group, while DMABPTSPd (PTSPd) did not (Figure 2A and 2B, **P < 0.01, ***P < 0.001, ****P < 0.0001).Meanwhile, the expression level of PCNA decreased in the two cell lines exposed to DMABTSPd(TSPd) (Figure 2A and 2B, *P < 0.05, **P < 0.01, ***P < 0.001, *vs* DMSO-group). Therefore, DMABTSPd(TSPd) could induce apoptosis of human gastric carcinoma cells and inhibit cell proliferation, while DMABPTSPd (PTSPd), with a similar structure to DMABTSPd (TSPd), could not. Additionally, Figure 2C showed that the expression level of cleaved-PARP and PCNA was not changed in human normal gastric mucosal epithelial cell line, Ges-1, exposed to different doses of DMABTSPd (TSPd) or DMABPTSPd (PTSPd) for 24 h, indicating that the two complexes had no obvious effect on cell apoptosis of human normal gastric mucosal epithelial cells.

### The involvement of a mitochondrial-related pathway in DMABTSPd(TSPd)-induced human gastric carcinoma cells apoptosis

**Figure 3:**
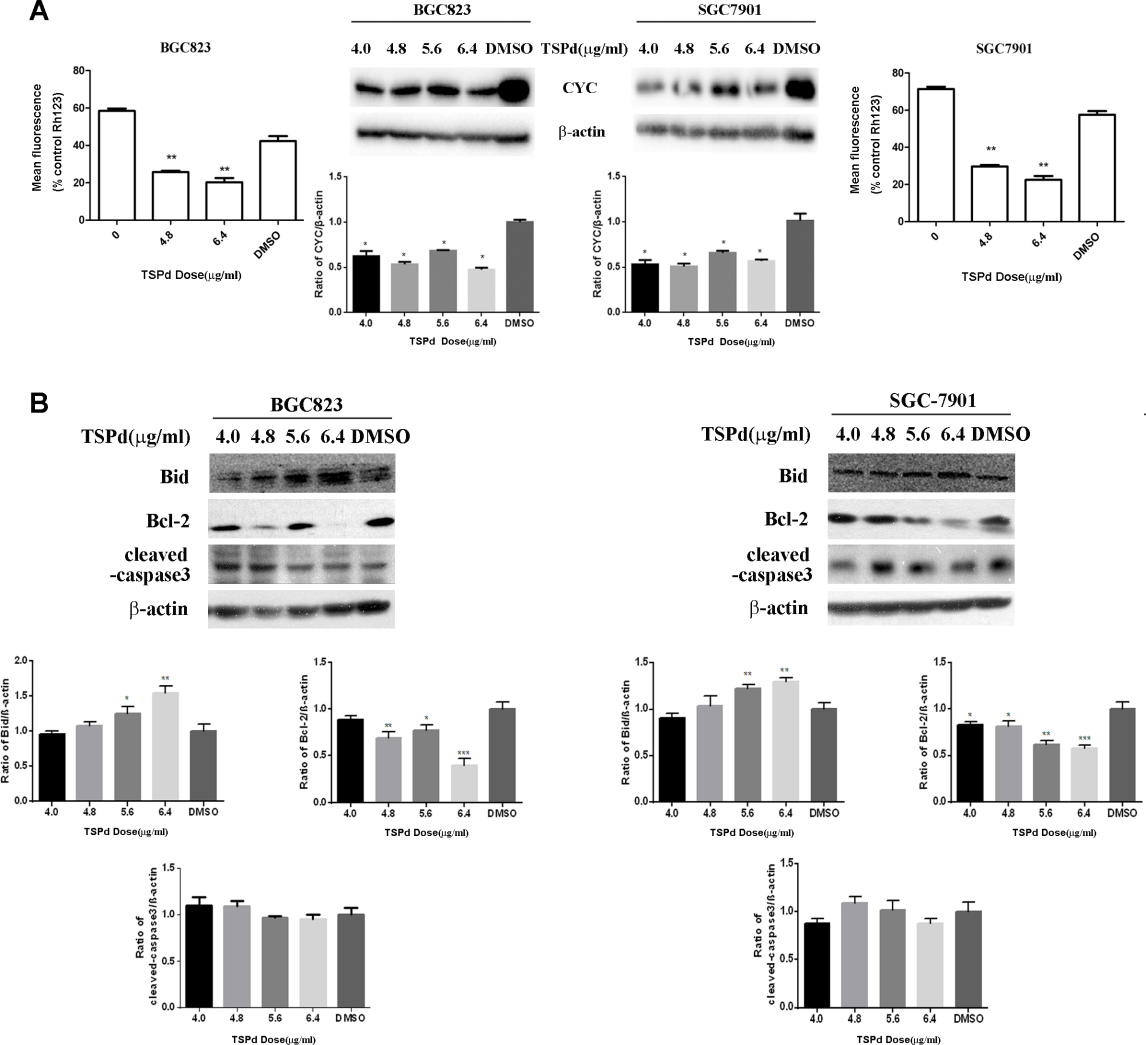
Effect of the target complexes on the mitochondrial signaling pathways in human gastric carcinoma cells **(A)** BGC823 cells and SGC7901 cells were collected after treated with different doses of DMABTSPd(TSPd) (0, 4.8, and 6.4 μg/mL) for 24 h and analyzed for mitochondrial integrity by Rh123 retention as described in the Materials and Methods section. Meanwhile, cells were collected after treatment with different doses of DMABTSPd(TSPd) (4.0, 4.8, 5.0, and 6.4 μg/mL) for 24 h, followed with the detection of cytochrome C(CYC) expression with western blotting as described in the Materials and Methods section. **(B)** BGC823 cells and SGC7901 cells were collected after treatment with different doses of TSPd (4.0, 4.8, 5.0, and 6.4 μg/mL) for 24 h, and the levels of Bid, Bcl-2, and cleaved-caspase 3 expression were detected by western blotting as described in the Materials and Methods section. Data are as means ± S.D. of three separate experiments (*P < 0.05, **P < 0.01, ***P < 0.001, vs the control group).

The mitochondria-mediated endogenous apoptosis pathway is one of the apoptosis pathways in mammalian cells. To investigate whether the regulation of DMABTSPd(TSPd) on apoptosis is associated with mitochondria, the mitochondria transmembrane potential (ΔΨM) of the two cell lines treated with different doses of DMABTSPd(TSPd) was measured by staining with a mitochondrial dye, Rho123 (Rhodamine123). Rho123 formed aggregates and emitted fluorescence that indicates an intact mitochondrial membrane potential in the control group. A significant decrease of Rho123 fluorescence was observed in the cells treated with different doses of DMABTSPd(TSPd), exhibiting that the membrane potential of these cells had been disrupted (Figure 3A, the outside panel, **P < 0.01, *vs* DMSO-group). The data then indicated that the DMABTSPd(TSPd)-induced apoptosis was associated with mitochondria transmembrane potential. Meanwhile, the expression of cytochrome c (CYC) was also measured with western blotting analysis. The results showed the significant decrease of CYC expression in DMABTSPd (TSPd)-treated BGC823 and SGC7901 cells, compared with DMSO-group(Figure 3A, the inner side panel, **P < 0.01), while there is no significant alteration of CYC expression in DMABPTSPd(PTSPd)-treated BGC823 and SGC7901 cells (Supplementary Figure 2).In addition, the two target complexes had not any effect on CYC expression in Ges-1 cells (Supplementary Figure 3).

The Bcl-2 family acts as major regulators of the mitochondrial apoptotic pathway. Within it, Bid may be the key regulator connecting the exogenous death receptor-mediated apoptosis pathway and the endogenous mitochondrial-mediated pathway, promoting the apoptotic signal transduction; Bcl-2 protein can bind the BH3 α helical region of pro-apoptotic proteins, inhibiting their pro-apoptotic effect. Thus, Bid and Bcl-2 are mutually antagonistic in the target cells. The expression levels of Bcl-2 and Bid were then detected using western blotting analysis. DMABTSPd(TSPd) led to the decrease of the expression level of Bcl-2 in a dose-dependent manner (Figure 3B, *P < 0.05, **P < 0.01, *vs* DMSO-group). Meanwhile, the expression level of Bid increased in a dose-dependent manner in the cells treated with different doses of DMABTSPd(TSPd) (Figure 3B,*P < 0.05, **P < 0.01,***P < 0.001, *vs* DMSO-group). Additionally, DMABTSPd (TSPd) had not any effect on the expression level of cleaved-caspase3 in BGC-823 and SGC-7901 cells. Therefore, the data showed that DMABTSPd(TSPd) may induce apoptosis via a mitochondria-related pathway in human gastric cancer cells, not associated with the activity of caspase3.

### The effect of the two complexes on tumor growth in a nude mouse tumor xenograft model derived from BGC-823 cells

**Figure 4:**
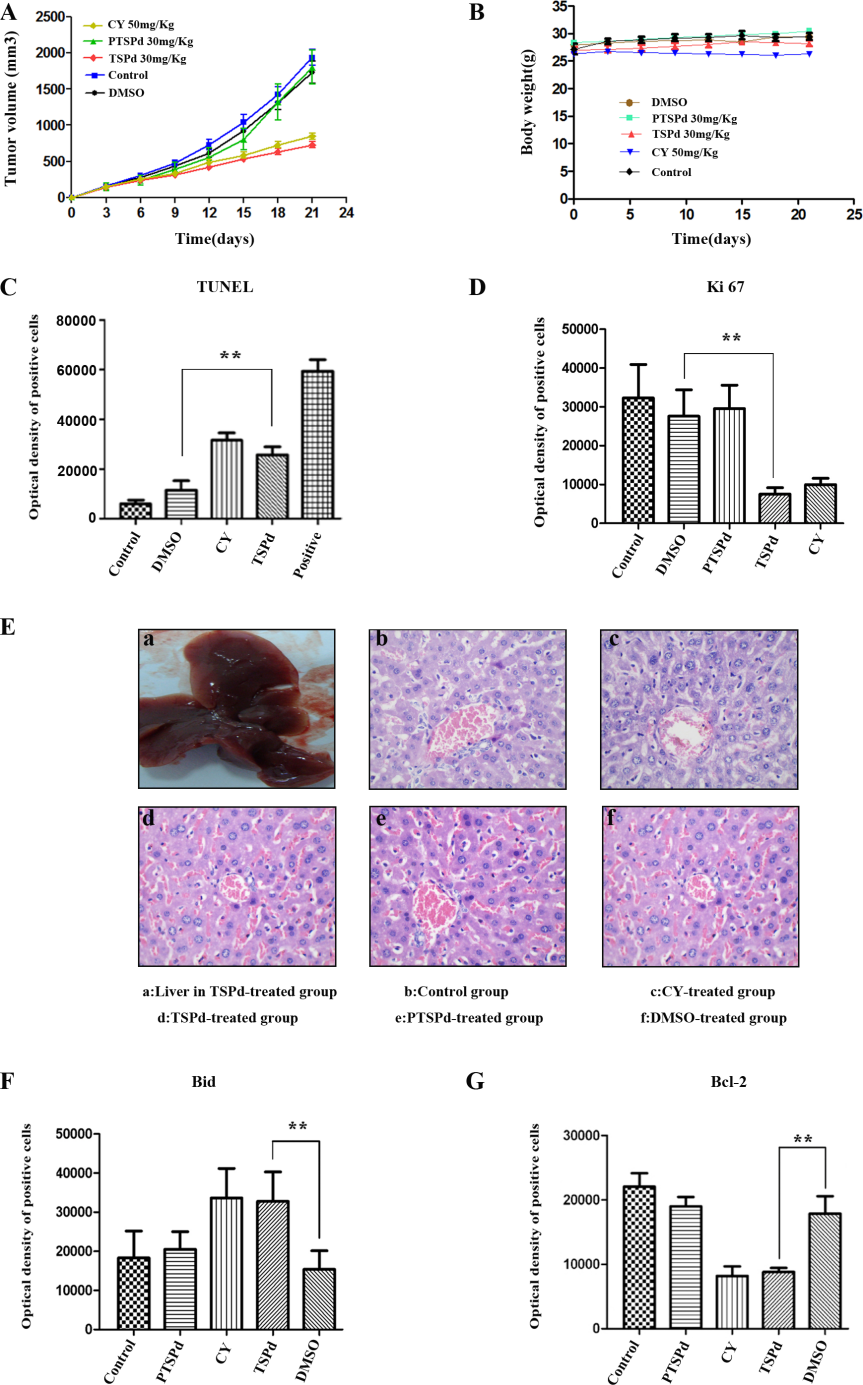
Effect of the target complexes on gastric cancer growth in a nude mouse tumor xenograft model Typical photographs of tumor samples from the nude mice treated with the target complexes (30 mg/kg), CY(50 mg/kg), DMSO, and vehicle control, respectively. **(A)** Variations in the volume of tumor samples from nude mice treated with the target and control complexes respectively. **(B)** Variations in the body weight of nude mice treated with the target and control complexes respectively. **(C)** The optical density of apoptotic cells of tumor samples from nude mice was detected using the TUNEL assay as described in the Materials and Methods section. (a. negative group(PBS); b. control group; c. DMSO-treated group; d. CY-treated group(positive); e. DMABTSPd(TSPd)-treated group; f. positive sample provided by Roche Diagnostics). **(D)** The level of Ki67 expression of tumor samples from nude mice was detected using immunohistochemistry. (a. negative group(PBS); b. control group; c. DMSO-treated group; d. DMABPTSPd (PTSPd)-treated group; e. DMABTSPd(TSPd)-treated group; f. CY-treated group(positive)). **(E)** The histological structure of liver from nude mice (a.General observation of liver from the DMABTSPd(TSPd)-treated group. (b–f): Liver stained by hematoxylin and eosin as described in the experimental section (b. control group; c. CY-treated group(positive); d. DMABTSPd(TSPd)-treated group; e. DMABPTSPd (PTSPd)-treated group; f. DMSO-treated group). F&G: The level of Bid **(F)** and Bcl-2 **(G)** expression was detected using immunohistochemistry. (a. negative group(PBS); b. control group; c. DMABPTSPd (PTSPd)-treated group; d. CY-treated group(positive); e. DMABTSPd (TSPd)-treated group; f. DMSO-treated group). Data are as means ± S.D. of each group (*P < 0.05, **P < 0.01, vs DMSO-treated group, magnification × 400).

To examine the antitumor efficacy of the two complexes, we investigated the tumor inhibition activity in a nude mouse model harboring tumor xenografts derived from the human gastric cancer cell line, BGC823. Remarkably, similar to the positive group (cyclophosphamide(CY)-treated group), the tumor volume in DMABTSPd(TSPd)-treated group decreased, compared with the control, DMSO, and DMABPTSPd (PTSPd)-treated group, without the alteration of the body weight of mouse (Supplementary Figure 4, Figure 4A, and Figure 4B). TUNEL assay is a common method for detecting DNA fragmentation resulting from apoptotic signaling cascades [15]. In the presence of horseradish peroxidase substrate diaminobenzidine (DAB), a very strong color reaction (dark brown) occurs specifically in apoptotic cells, thus apoptotic cells can be observed and counted under Olympus X41 microscope. The results of TUNEL assay showed that DNA damage had occurred significantly in the tumor tissue sections of the DMABTSPd(TSPd)-treated group (Supplementary Figure 5A and Figure 4C, **P < 0.01, *vs* DMSO-group), indicating that DMABTSPd(TSPd) led to cell apoptosis in these nude mice. Meanwhile, DMABTSPd(TSPd) reduced the expression of Ki67 using immunohistochemical staining (Supplementary Figure 5B and Figure 4D, **P < 0.01, *vs* DMSO-group), which is an important index refection of cell proliferation. Additionally, the hematoxylin and eosin staining showed that the liver in DMABTSPd(TSPd)-treated nude mice appeared to have a normal anatomic and histological structure, indicating that DMABTSPd(TSPd) suppressed gastric carcinoma growth without liver damage in a nude mouse tumor xenograft model (Figure 4E). Therefore, DMABTSPd(TSPd) exhibited an inhibitory effect on tumor growth in a nude mouse tumor xenograft model.

To further investigate whether the mechanism of DMABTSPd(TSPd) inhibition on tumor growth in a nude mouse tumor xenograft model is correlated with mitochondrial pathways, similar to the result *in vitro*, the expression level of Bid and Bcl-2 was detected using immunohistochemistry. The results show that DMABTSPd(TSPd) led to the increase at Bid level (Supplementary Figure 6A and Figure 4F, **P < 0.01, *vs* DMSO-group), and the decrease at Bcl-2 level in a nude mouse tumor xenograft model (Supplementary Figure 6B and Figure 4F, **P < 0.01, *vs* DMSO-group). Therefore, the inhibition of DMABTSPd(TSPd) on tumor growth in a mouse tumor xenograft model may be associated with mitochondria-related pathway.

## DISCUSSION

This study demonstrates for the first time that a *trans*-thiosemicarbazone-based, Schiff base palladium (Pd) (II) complex, DMABTSPd(TSPd) (with the bare amino group), could induce apoptosis in human gastric carcinoma cells *in vitro* and suppress tumor growth in a nude mouse tumor xenograft model derived from BGC-823 cells via a mitochondrial-related pathway, without obvious side effect on normal human gastric epithelial cell and mouse liver. The data indicated that DMABTSPd(TSPd) exhibited a significant antitumor effect in human gastric adenocarcinoma cells. However, another similar *trans*-thiosemicarbazone-based, Schiff base palladium (Pd) (II) complex, DMABPTSPd (PTSPd) (without the bare amino group), did not have any antitumor activity. It is suggested that DMABTSPd(TSPd) is worthy of further structural optimization and representing a promising Pd complex for the development of a new antitumor therapeutic agent.

Several strands of evidence show that Pd complexes have significant anti-cancer activity and anti-drug resistance compared with Pt chemistry [16–19]. As an example, Pd (II) saccharinate complexes with bis (2-pyridylmethyl) amine induce cell death by apoptosis in human breast cancer cells *in vitro* [17]. A Pd (II)-saccharinate complex containing terpyridine powerfully inhibits the growth of fibrosarcoma cells by inducing apoptosis [18]. In gastric cancer cells, [PdCl2 (L)] is a potent chemotherapeutic agent for Cisplatin-resistant gastric cancer and may have clinical applications [19]. Here, it is demonstrated that the *trans*-thiosemicarbazone-based, Schiff base palladium (Pd) (II) complex, DMABTSPd(TSPd) (with the bare amino group), could inhibit the cell viabilities, promote the cleavage of PARP, and reduce the expression of PCNA, suggesting that DMABTSPd(TSPd) induces apoptosis in human gastric carcinoma cell lines, BGC823 and SGC7901, consistent with the above studies. Furthermore, it is observed that DMABTSPd(TSPd) could suppress tumor growth in a mouse tumor xenograft model derived from BGC-823 cells, including the decrease of tumor volume and cell proliferation, and the increase of apoptotic cells. Similarly, a Pd(II) complex, [Pd(sac)(terpy)](sac)·4H_2_ O, has also been reported to exhibit a strong anticancer activity against Ehrlich ascites carcinoma by inducing apoptosis and suppressing proliferation *in vivo* [20]. Especially, DMABTSPd(TSPd) did not have obvious effect on normal human gastric epithelium cell, GET-1, *in vitro*, and did not damage the morphological characteristics of liver in nude mouse model, indicating that DMABTSPd(TSPd) did not exhibit unequivocal side-effect. Therefore, the data that DMABTSPd(TSPd) has a significant antitumor activity in BGC823 and SGC701 cells leads to the possibility that it becomes a new antitumor therapeutic agent.

**Figure 5:**
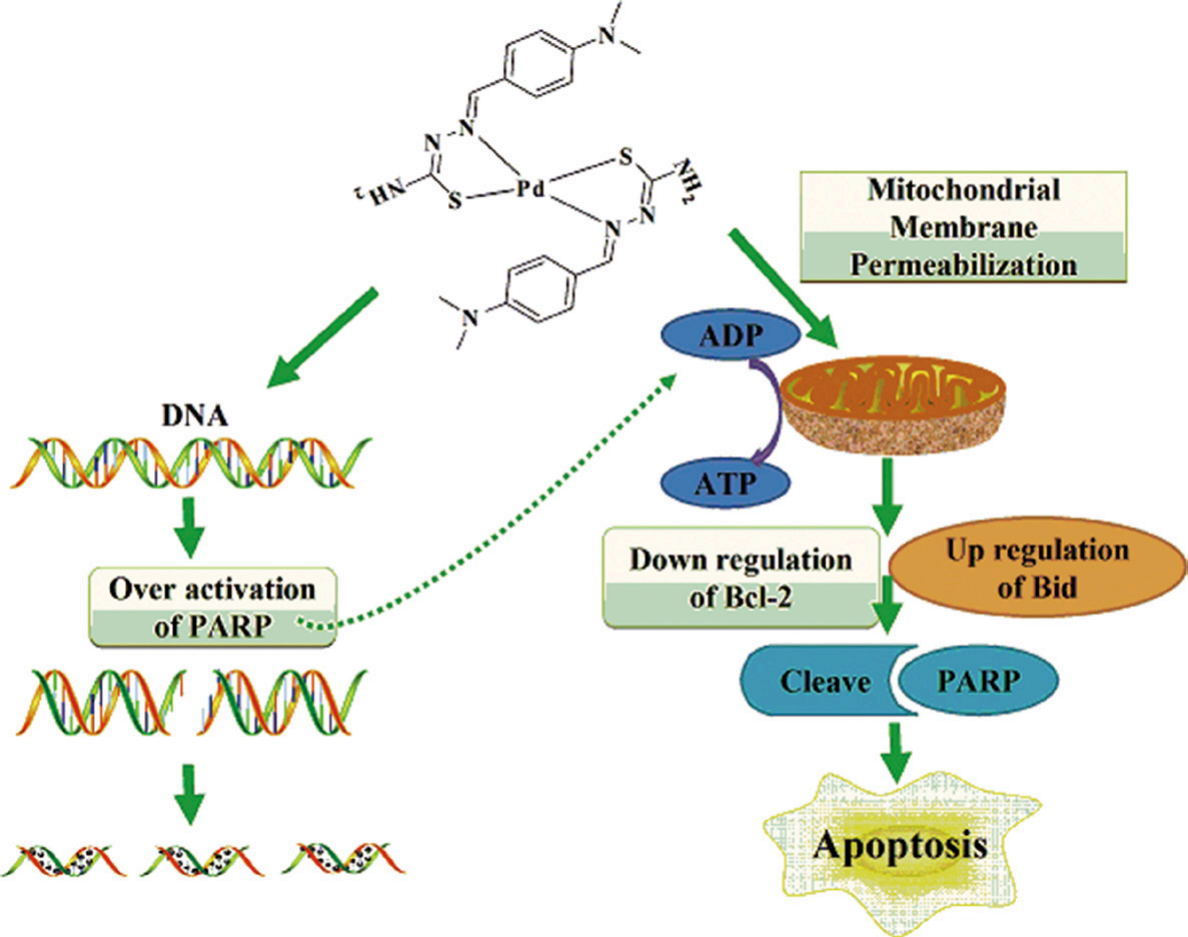
Molecular mechanism of DMABTSPd(TSPd)-induced apoptosis in human gastric carcinoma cells

Results presented in this manuscript showed that the inner mitochondrial membrane potential decreased with increasing drug dose of DMABTSPd(TSPd). Meanwhile, the expression level of Bcl-2, one of anti-apoptotic members, reduced with increasing drug dose of DMABTSPd(TSPd). In contrast, the expression level of Bid, one of pro-apoptotic members, increased significantly with increasing drug dose of DMABTSPd(TSPd). Similar results were detected in a nude mouse tumor xenograft model derived from BGC-823 cells. These results show that DMABTSPd(TSPd)-induced apoptosis in human gastric cancer cells is largely dependent on the endogenous mitochondrial pathway and that the regulation protein molecules mainly include the Bcl-2 and Bid of the Bcl-2 family members. The regulatory mechanism of other Pd complexes associated with mitochondrial pathway has also been reported in prostate cancer cells. For example, a new ionic Pd(II) complex, [(bipy)Pd(Pcurc)][CF_3_SO_3_], with the metal center coordinated to two different chelating ligands, the pure curcumin (Pcurc) and the 4,4′-dinonyl-2,2′-bipyridine (bipy), induces both cell growth inhibition and apoptosis of human prostate cancer cells, (LnCaP, PC3, and DU145) through the production of ROS and JNK phosphorylation associated with GSTp1 down-regulation. ROS production induced by the Pd complex treatment activated apoptosis through mitochondrial membrane depolarization in all prostate cancer cells, with up-regulation of Bax and down-regulation of Bcl-2 proteins [21]. On the other hand, caspase activation is thought to be important for PARP-related cell apoptosis. However, some reports have shown that PARP-dependent toxicity appears to be caspase-independent [22, 23]. It is implied that DMABTSPd(TSPd)-induced apoptosis may also be in a caspase-3-independent manner in human gastric cancer cells. Overall, the mitochondrial pathway could be involved in regulating cell apoptosis of Pd complex in gastric adenocarcinoma cells. Therefore, the mechanism by which DMABTSPd(TSPd) induced gastric cells apoptosis may be because: DMABTSPd(TSPd) could change the mitochondrial membrane permeability through reducing the mitochondrial transmembrane potential and adjusting the expression of Bcl-2 and Bid, resulting in apoptosis; DMABTSPd(TSPd) treatment not only causes excessive activation of PARP-1, leading to intracellular ATP/NAD depletion, which influences mitochondrial membrane potential, but also initiates the mitochondrial-related apoptosis pathway. The two processes exist in synergy to induce apoptosis (Figure 5).

The biological activity of transition metal complexes formed with different thiosemicarbazone derivatives varies widely, and different transition metal complexes formed with the same thiosemicarbazone derivatives show significant differences in antibacterial and antitumor activities. Even a slight change in the ligand substituents while keeping the metal center the same can have a dramatic effect on the biological activity. In this study, DMABPTSPd (PTSPd), with a phenyl substituent on the amino group, does not have such activity. The differences in cytotoxicities between the two complexes might be related to structural steric hindrance for the DMABPTSPd (PTSPd) complex. DMABPTSPd (PTSPd) with two phenyl substitutents may have more steric hindrance than DMABTSPd(TSPd). The molecular size of DMABTSPd(TSPd) is smaller than that of DMABPTSPd (PTSPd). It might be that these two amine groups will probably help the Pd complex to reach its cellular target. In most of the thiosemicarbazone-based Schiff base Pd (II) complexes, the ligands are usually bound to the Pd (II) center with an O, N, S-tridentate coordination mode. This tridentate coordination mode stabilizes the Pd (II) complexes and prevents any *cis-trans* isomerization to give an enhanced effect [11]. The tridentate coordination mode of the Pd (II) complex is neither *cis*-geometry nor *trans*-geometry. The importance of the *trans*-geometry around the Pd center has been attributed to the comparatively higher cytotoxicity values compared with those for *cis*-isomers. Our designed title complexes are simply *trans*-geometry thiosemicarbazone-based Schiff base Pd (II) complexes. It is apparent from the data presented over the years of reasearch into metallopharmaceutical agents that both the metal and the ligand determine the biological activity [1–4]. A suitable thiosemicarbazone carrier ligand may be crucial for the efficacy against tumors because they play an important role in stabilizing the metal ion, imparting substituents inertness, modifying reactivity and other positive impacts on the target site. DMABPTSPd (PTSPd), where the only difference in structure compared with DMABTSPd(TSPd) is that the amino group is substituted with a phenyl group, inhibition effect on cell viability is not obvious. Additionally, BGC823 and SGC7901 cells are especially sensitive to DMABTSPd(TSPd) with IC_50_ values of 5.1 ug/mL and 5.4 ug/mL, respectively, compared with DMABPTSPd (PTSPd). It seems that the amino group plays an important role in the differences in cytotoxicity and sensitivity to cancer cells between the two complexes.

## CONCLUSIONS

In summary, our study has investigated the antitumor activity and molecular mechanism of the apoptosis of DMABTSPd(TSPd), a potential antitumor therapeutic agent. Further optimization of its chemical structure and DMPK properties, such as water-solubility, could ultimately provide a new, low-toxicity, efficient, and highly targeted antitumor drug. Interestingly, compared with DMABTSPd(TSPd), the phenyl-substituted analog DMABPTSPd (PTSPd) is unable to effectively inhibit the growth of BGC823 and SGC7901 human gastric cancer cells. It seems that the amino group plays an important role in the different cytotoxicity of these two complexes. Our study provides an example of how small changes in molecular structure could lead to profound differences in biological activity.

## MATERIALS AND METHODS

### Reagents and antibodies

The two *trans*-thiosemicarbazone-based, Schiff base palladium (Pd)(II) complexes were prepared and characterized as described previously in our group [24, 25]. Antibodies against β-actin, Bcl-2, PARP, Ki67, Bid, PCNA, and Caspase-3 were purchased from Santa Cruz Biotechnology, Inc. (Santa Cruz, CA, USA). The other common reagents used were all commercially available.

### Cell culture and treatment with the two complexes

The human gastric cancer cell lines BGC-823 and SGC7901, and normal human gastric mucosal epithelial cell line Ges-1 were obtained from the Shanghai Institute of Cell Biology, Chinese Academy of Sciences (Shanghai, China), and were maintained in RPMI1640 supplemented with 10% FBS, 100 U/mL penicillin, and 100 μg/mL streptomycin, at 37°C in a water-saturated atmosphere of 5% CO_2_. Cells were treated by the two drugs as indicated dose or time point, respectively, with the highest dose DMSO as the control group.

### General procedures for IC_50_ determination of the target complexes

Cells were plated in 96-well plates (3 × 10^3^ cells/well). After 24 h, the cells were treated with drugs or DMSO at the indicated doses in centuplicate. 20 μl MTT reagent was added to the wells and incubated at 37°C for 4 h before and after the treatment period of 48 h. The media was then discarded gently and 150 μL DMSO was added, followed by the incubation at room temperature for 15 min and the detection at 570 nm. The initial rate data collected were used for determination of IC_50_ values. For IC_50_ determination, kinetic values were obtained directly from nonlinear regression of substrate–velocity curves in the presence of various doses of inhibitor using one-site competition in GraphPad Prism 6 scientific graphing software [26, 27].

### Cell viability assay

Cells were plated in 96-well plates (3 × 10^3^ cells/well). After 24 h, the cells were treated with drugs or DMSO at the indicated doses, followed by an MTT (3-(4,5-dimethylthiazol-2-yl)-2,5-diphenyltetrazolium bromide, MTT) assay [28]. The product was then dissolved in dimethylformamide and quantified spectrophotometrically at a wavelength of 490 nm with a reference wavelength of 630 nm as described previously [28, 29]. The OD values correspond to the number of viable cells.

### Mitochondria transmembrane potential (ΔΨM)

Cells were resuspended in 100 μL of 5 μM Rhodamin123 staining buffer. After incubation at 37°C for 30 min, cells were immediately subjected to flow cytometry analysis [26, 27].

### Western blotting analysis

Protein extracts were electrophoresed on 8–12% denaturing gel and electroblotted onto nitrocellulose membrane. The membrane was incubated with various antibodies as required at 4°C overnight, followed by the addition of the corresponding secondary antibody at room temperature for 3–4 h. An ECL kit (Pierce, Rockford, IL,USA) was used to detect the antibody reactivity [28, 29].

### Xenograft assay in nude mice

Fifty 6-week-old female BALB/Cnu/nu nude mice were purchased from Shanghai SLAC Laboratory Animal Co. Ltd (Shanghai, China). All animal studied were fed until each body weight were higher than 25 g and then were conducted according to the regulations of the IACUC protocol. The study was approved by the Committee on the Ethics of Animal Experiments of the University of Xiamen (ID No. 20130916). 200 μl BGC-823 cells (2 × 10^6^/mouse) in culture medium were subcutaneously injected into the left flank of the mouse. Animals bearing tumors were randomly assigned to five groups; vehicle control group (double-distilled water), cyclophosphamide(CY) (purity 100%; Sigma-Aldrich, Shanghai, China) group, DMSO group, DMABTSPd(TSPd) group, and DMABPTSPd (PTSPd) group with 10 mice per group. After a 72-h injection of BGC-823 cells, mice were injected intratumorally with indicated agents once every 3 days for 21 days [28]. Tumor volume was measured and analyzed using the SAS program PROCMIXED during this period. All mice were killed and examined for the growth of subcutaneous tumors at 24 h after stopping the injection.

### Immunohistochemistry

The fresh tumor samples were fixed in 4% paraformaldehyde for 48 h and then paraffin embedded for further routine histological preparation. Four-micrometer-thick sections were deparaffinized in xylene and rehydrated in graded alcohols and distilled water. After antigen retrieval, endogenous peroxidase activity was blocked with 3% hydrogen peroxide in methanol for 10 min at room temperature followed by rehydration in PBS, and incubation with 10% goat serum for 10 min to bind nonspecific antigens. As described in the manufacturer's instructions (MAIXIN.BIO, Fuzhou, China), the sections were incubated overnight at 4°C with Ki67, Bcl-2, and Bid (1:100 dilutions) primary antibody and, subsequently, with secondary antibody (1:400) for 60 min at room temperature. Diaminobenzidine was used to visualize the immunohistochemical reaction followed by counterstaining with hematoxylin. In accordance with previous studies [30], the average optical density (OD) of positive cells was measured and analyzed using an ImagePro Plus 6.0 system.

### Hematoxylin and eosin staining

Fresh liver samples from the five groups were observed and fixed in 4% paraformaldehyde for 48 h, and then paraffin embedded for further routine histological preparation. Five-micrometer-thick sections were deparaffinized in xylene and rehydrated in graded alcohols and distilled water, followed by hematoxylin and eosin staining.

### Tissue TUNEL assay

For *in situ* visualization of apoptotic cells, the TUNEL assay was performed in tumor tissue according to the manufacturer's instructions with *in Situ* Cell Death Detection Kit, POD (Roche Diagnostics, Shanghai, China). In TUNEL staining, dark brown cells were considered to be positive and were counted throughout microscopically magnified fields (magnification ×100) of each tissue section. The percentage of positive cells was analyzed by SPSS v.15.0 for Windows [30].

### Statistical analysis

Data were expressed as means ± S.D. of at least three independent experiments, and statistical analysis for single comparison was performed using the Student's *t* test. The criterion for statistical significance was *p* < 0.05.

### Abbreviations

DMABTSPd (TSPd) ; DMABPTSPd (PTSPd); Dimethyl Sulphoxide(DMSO); cyclophosphamide(CY); diaminobenzidine(DAB); 3-(4,5-dimethylthiazol-2-yl)-2,5-diphenyltetrazolium bromide (MTT); transferase-mediated deoxyuridine triphosphate-biotin nick end labeling(TUNEL); poly ADP-ribose polymerase(PARP); proliferating cell nuclear antigen(PCNA); cytochrome C(CYC); half maximal inhibitory concentration(IC50).

## SUPPLEMENTARY MATERIALS FIGURES AND TABLES


