# Supplementary material for: Antitumor activity of a Trans-thiosemicarbazone schiff base palladium (II) complex on human gastric adenocarcinoma cells
Source: Oncotarget. 2017 Jan 13;8(8):13620–31. doi: 10.18632/oncotarget.14620 (PMC5355125; doi:10.18632/oncotarget.14620)
Supplement: Supplementary file 1 [file oncotarget-08-13620-s001.pdf]

Antitumor activity of a *Trans*-thiosemicarbazone schiff base palladium (II) complex on human gastric adenocarcinoma cells

Supplementary Materials

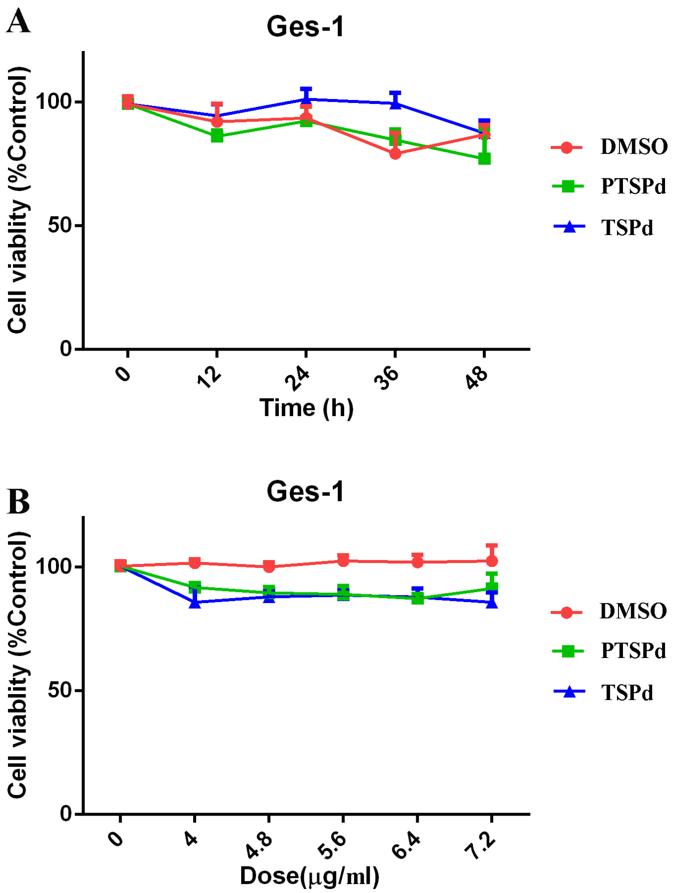

Supplementary Figure 1: The effect of the target complexes on the cell viability of normal human gastric epithelial cell line, Ges-1. (**A**) Cells were treated with different doses of target complexes (0, 4.0, 4.8, 5.6, 6.4, and 7.2 μg/mL) and DMSO (the same volume as the highest drug dose). (**B**) Cells were treated with 6.4 μg/mL target complexes for different time periods (0, 12, 24, 36, and 48 h). The cell viabilities were measured by an MTT assay as described in the Materials and Methods section.

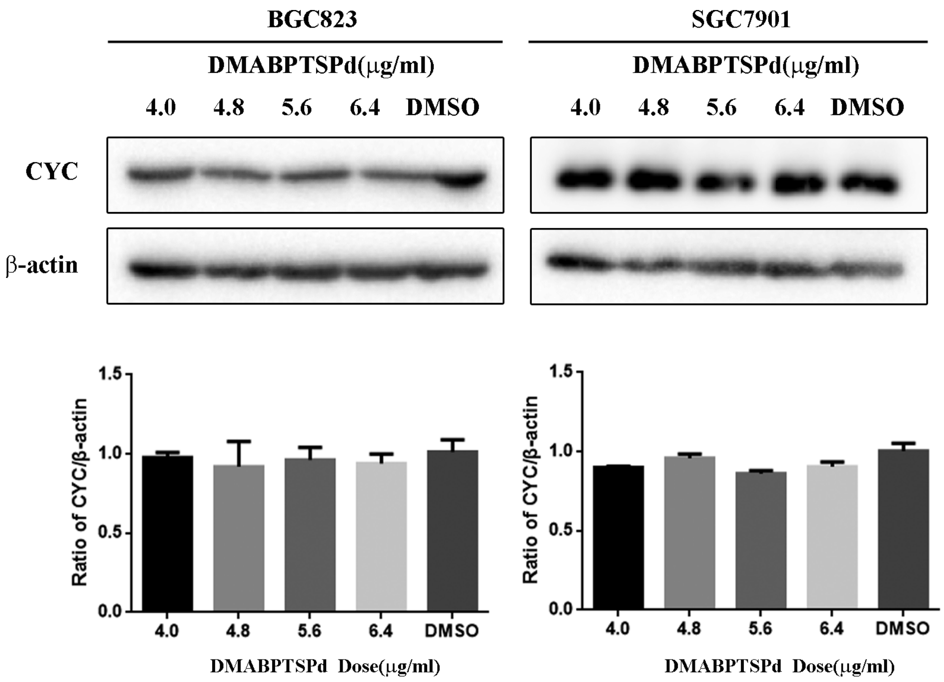

Supplementary Figure 2: The effect of DMABPTSPd(PTSPd) on cytochrome C expression in human gastric carcinoma cells. BGC823 and SGC7901 cells were collected after treatment with different doses of DMABPTSPd(PTSPd) (4.0, 4.8, 5.0, and 6.4 μg/mL) for 24 h, followed with the detection of cytochrome C(CYC) expression with western blotting as described in the Materials and Methods section.

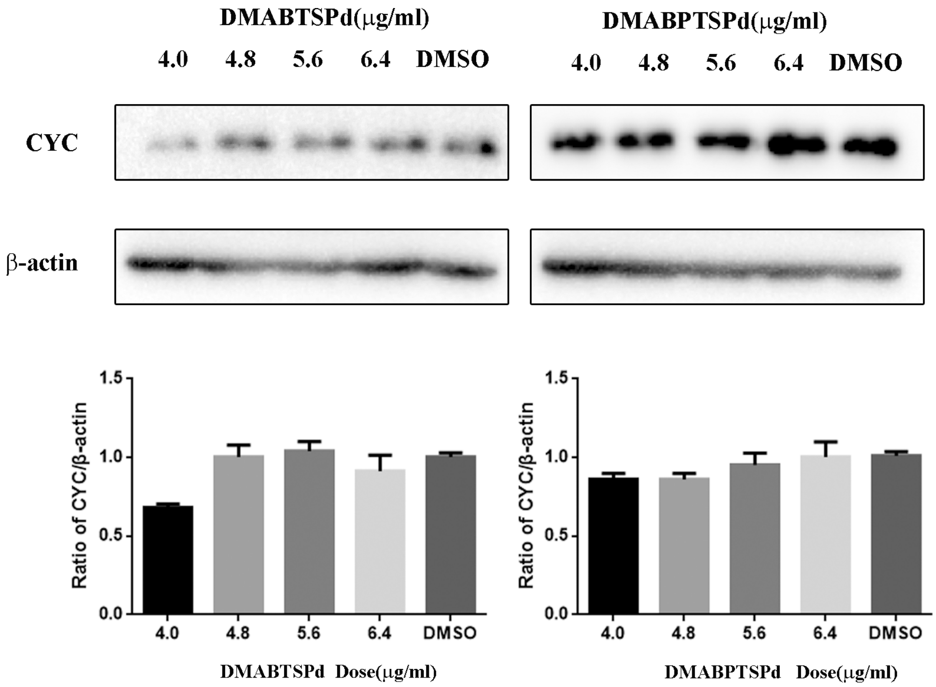

Supplementary Figure 3: The effect of the two target complexes on cytochrome C expression in normal human gastric mucosal epithelial cells. Ges-1 cells were collected after treatment with different doses of DMABTSPd(TSPd) or DMABPTSPd (PTSPd) (4.0, 4.8, 5.0, and 6.4 μg/mL) for 24 h, followed with the detection of cytochrome C(CYC) expression with western blotting as described in the Materials and Methods section.

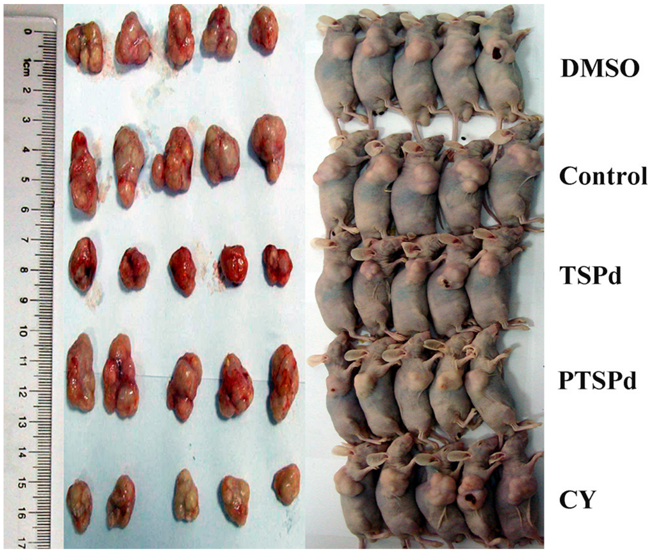

Supplementary Figure 4: Morphological variations in tumor samples from nude mice treated with the target and control complexes respectively.

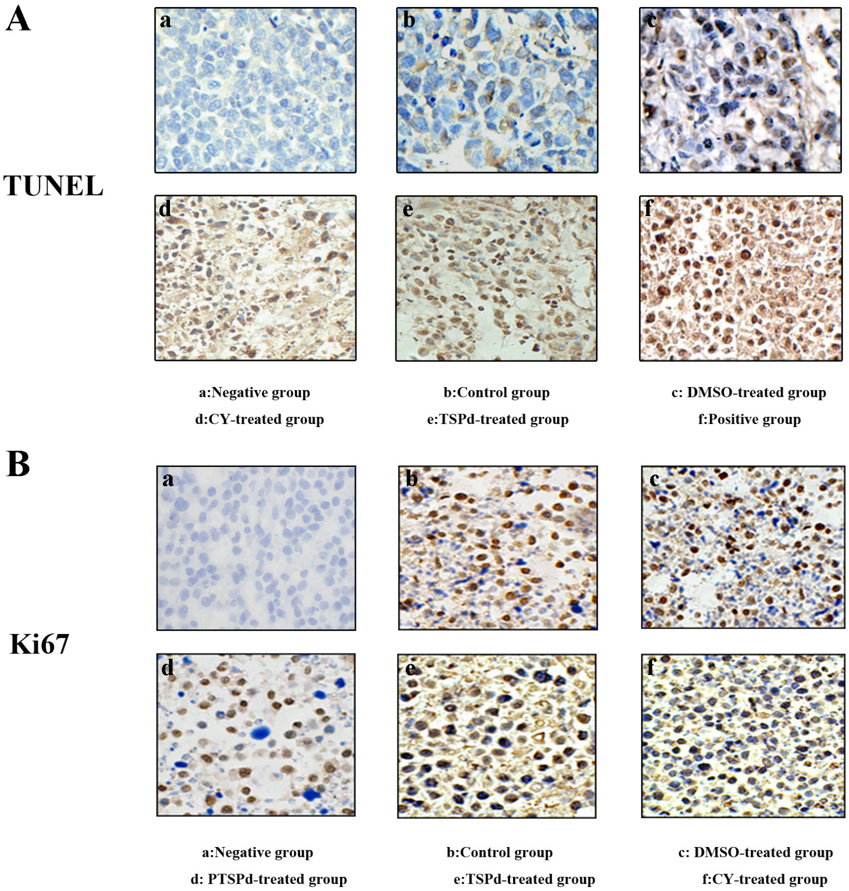

Supplementary Figure 5: The apoptotic cells and proliferative cells were detected in tumor samples from nude mice treated with the target and control complexes respectively. (**A**) The optical density of apoptotic cells of tumor samples from nude mice was detected using the TUNEL assay as described in the experimental section. (a. negative group(PBS); b. control group; c. DMSO-treated group; d. CY-treated group(positive); e. DMABTSPd(TSPd)-treated group; f. positive sample provided by Roche Diagnostics, magnification ×400). (**B**) The level of Ki67 expression in tumor samples from nude mice was detected using immunohistochemistry. (a. negative group(PBS); b. control group; c. DMSO-treated group; d. DMABPTSPd (PTSPd)-treated group; e. DMABTSPd(TSPd)-treated group; f. CY-treated group(positive), magnification ×400).

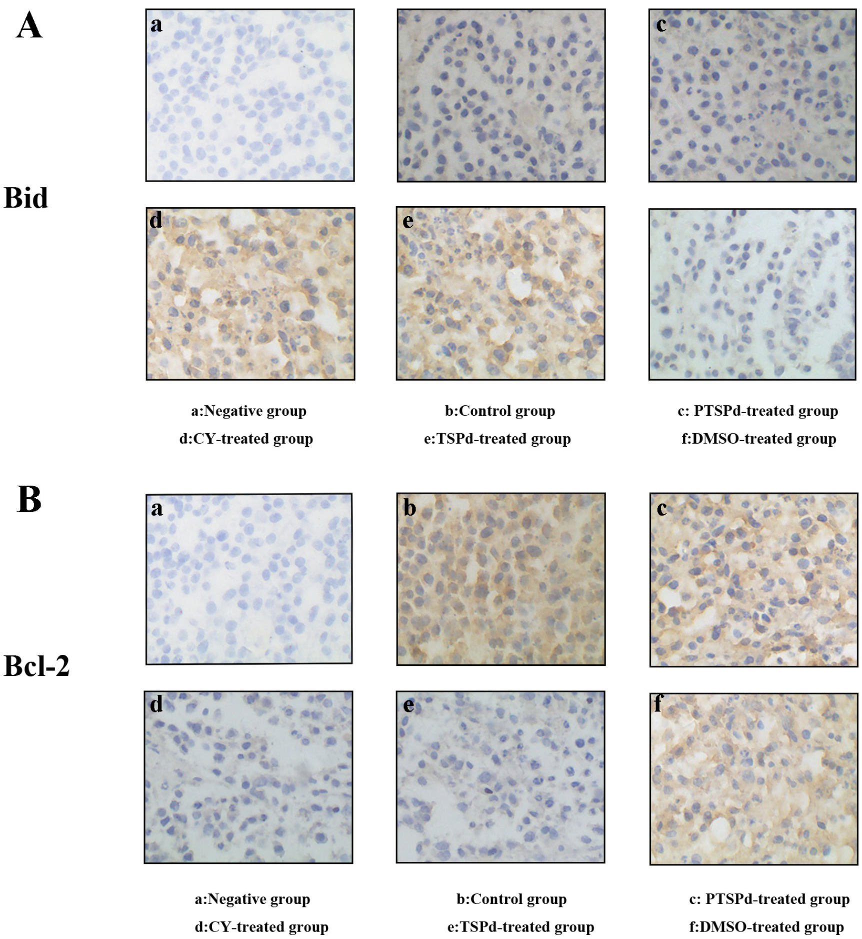

Supplementary Figure 6: The levels of Bcl-2 (**A**) and Bid (**B**) expression in tumor samples from nude mice were detected using immunohistochemistry. (a. negative group(PBS); b. control group; c. DMABPTSPd (PTSPd)-treated group; d. CY-treated group(positive); e. DMABTSPd(TSPd)-treated group; f. DMSO-treated group).
